# Global Burden and Trends in Incidence, Mortality, and Disability of Stomach Cancer From 1990 to 2017

**DOI:** 10.14309/ctg.0000000000000406

**Published:** 2021-10-05

**Authors:** Yuheng Qin, Xin Tong, Jiahui Fan, Zhenqiu Liu, Renjia Zhao, Tiejun Zhang, Chen Suo, Xingdong Chen, Genming Zhao

**Affiliations:** 1 Key Laboratory of Public Health Safety (Fudan University), Ministry of Education, China;; 2 Department of Epidemiology, School of Public Health, Fudan University, Shanghai, China;; 3 School of Public Health, Zhejiang University, Hangzhou, Zhejiang, China.; 4 State Key Laboratory of Genetic Engineering and Collaborative Innovation Center for Genetics and Development, School of Life Sciences, Fudan University, Shanghai, China;; 5 Fudan University Taizhou Institute of Health Sciences, Taizhou, China;; 6 Human Phenome Institute, Fudan University, Shanghai, China;

## Abstract

Stomach cancer is a serious global public health problem. The current burden of stomach cancer and its trends across time and location need to be understood to develop effective preventive strategies.

**METHODS:**

Data were obtained from the Global Burden of Disease study. The burden of stomach cancer and variations in time and geographical regions were assessed according to the age-standardized rate and estimated annual percentage change (EAPC) of the incidence and mortality rate between 1991 and 2017. We also investigated the associations between the relevant rates and sociodemographic index (SDI).

**RESULTS:**

Overall, the age-standardized incidence rate (EAPC = −1.36, 95% confidence interval [CI]: −1.47 to −1.25), age-standardized mortality rate (EAPC = −2.2, 95% CI: −2.29 to −2.12), and age-standardized disability-adjusted life years rate (EAPC = −2.52, 95% CI: −2.63 to −2.43) decreased worldwide from 1990 to 2017. This trend varied across different countries and regions and according to sex and age. SDI had a significant negative correlation with the age-standardized mortality rate (*P* < 0.01, r = −0.28) and age-standardized disability-adjusted life years rate (*P* < 0.01, r = −0.31). Similar negative correlations were observed between SDI and the EAPC.

**DISCUSSION:**

The observed correlation between SDI and disease burden suggests that strategically implementing the screening and eradication of *Helicobacter pylori*, improving the medical level in countries with low SDI, and promoting the implementation of tobacco cessation policies would help reduce the disease burden of stomach cancer.

## INTRODUCTION

According to GLOBOCAN 2018 (1), stomach cancer has the fifth highest incidence rate among all malignant tumors globally and is the third most common cause of mortality. It is one of the most common tumors worldwide. In 2017, the global incidence of stomach cancer was 1,220,662 (95% uncertainty interval [UI] = 1,189,032–11,254,563) and the associated mortality was 864,989 (95% UI = 848,254–884,655), resulting in 19,130,771 (95% UI = 18,738,585–19,569,400) disability-adjusted life years (DALY) (2,3). The incidence and mortality of stomach cancer varied in different regions in 2017. Although the incidence of stomach cancer has declined in the past century, it accounts for a significant part of the global disease burden (2).

The 2 main tumor sites of stomach adenocarcinomas are proximal (cardiac) and distal (noncardiac) (4). The incidence of noncardiac stomach cancer has been declining in the past decades, mainly because of the steady decline in *Helicobacter pylori* infection (5). The rate of *H. pylori* infection has decreased in many countries because of not only improvements in their social and economic conditions in recent years but also use of antibiotics and better medical treatment (6). In addition, eating vegetables and fruits and performing physical exercise are protective factors for stomach cancer (3,7). Despite this, smoking, a high-sodium diet, and *H. pylori* infection are considered risk factors for stomach cancer (3,7).

Previous studies described variations only in trends of stomach cancer without conducting quantitative analyses. Our study used data from the Global Burden of Disease (GBD) study based on the age-standardized rate (ASR) of stomach cancer in 195 countries and territories to analyze: (i) variations in changing trends in the burden of stomach cancer according to age and sex and (ii) the relationship between the ASR in stomach cancer and these trends and sociodemographic index (SDI) to provide a scientific reference point to study the etiology of stomach cancer and for the development of early prevention strategies and measures.

## METHODS

### Data sources

Data on the age-standardized incidence rate (ASIR), age-standardized mortality rate (ASMR), and age-standardized disability-adjusted life rate (ASDR) for stomach cancer according to sex and age were collected using the Global Health Data Exchange Query Tool (http://ghdx.HealthData). We collected data for 195 countries and territories from 1990 to 2017. We divided these 195 countries and territories into 21 regions by geographical location and 5 regions by SDI level (including low, low-middle, middle, high-middle, and high). The Global Health Data Exchange query tool was created by the Institute for Health Metrics and Evaluation to share the available health data resources with global health researchers. The GBD 2017 database and the method for estimating the burden of stomach cancer have been described in detail previously (8,9).

### Disease measurement index and SDI

This study used the incidence, mortality, and DALY rates of stomach cancer to represent the burden of stomach cancer. The incidence rate is defined as the rate of new cases of a disease during a certain period within a certain range of people (10). The mortality rate is the proportion of deaths in a given time and range (10). DALY estimates all healthy life years lost from comorbidity onset to death, including years of life lost due to early death and disability due to disease loss of healthy life (years lived with disability) (10). All disease indicators were adjusted according to the standard age and calculated using the direct method, with the world standard population as the standard unit (11). SDI data from 195 countries were collected from a previous study of GBD 2017 (9). SDI is a composite indicator of development status heavily related to health outcomes, which can be calculated based on the income per capita, average educational attainment, and total fertility rates of a population (9). In detail, it is the geometric mean of total fertility before 25 years, mean education of persons aged 15 years and older, and lag-distributed income per capita, with values ranging from 0 to 1 (9). An SDI value of 0 indicates that the development level relevant to health in that location is the lowest. By contrast, when the SDI value is 1, the development level relevant to health is the highest.

### Age-standardized rate

The ASIR, ASMR, and the ASDR were used to quantify the burden of stomach cancer. It is necessary to standardize the index of stomach cancer when comparing the burden of stomach cancer in different age groups or at different periods in the same population. The ASR (per 100,000 people) was calculated using the direct method, based on the world standard population (11). The following calculation formula was used:

$$\text{ASR}=\frac{\sum_{i=1}^{n}aiWi}{\sum_{i=1}^{n}Wi},$$

where *i* represents different age groups, *a* represents the incidence, mortality, or DALY rate in specific age groups, and *w* represents the number of people in each age group in the standard population.

### Estimated annual percentage change

The changing trends in the ASR can provide clues about changes in the risk factors and disease patterns of stomach cancer in the population. To further understand the changing trends in the global burden of stomach cancer, we calculated the estimated annual percentage change (EAPC) of the ASR for stomach cancer to quantitatively analyze these changing trends.

Calendar year was used as the explanatory variable *X* when calculating the EAPC, and the logarithm (ln [ASR]) based on the natural number of the ASR was used as the dependent variable *Y* to fit the data to the regression line *y* = *a* + *bx* + *ε*. Subsequently, we used the fitted regression line parameter *β* to calculate the EAPC as follows: EAPC = 100 × (exp(*β*) − 1). The EAPC can only be calculated when ASR changes remain stable throughout the observation period. Statistical hypothesis testing is required to assess the calculated EAPC to exclude the influence of random factors. The hypothesis test for the EAPC is equivalent to the hypothesis test for the slope of a fitted line, i.e., if the slope of the line is statistically significant, the EAPC is considered effective. The hypothesis test for EAPC is a *t* test of slope *b* of the fitted line: *tb* = *b*/sb (*b* is the slope of the line and sb is the SE of slope *b*), and the degrees of freedom, *V*, is the number of calendar years minus 2. Because of the influence of the SE of slope *b* on the slope of the fitted line and EAPC, the 95% confidence intervals (CIs) of the EAPC need to be calculated (12) as follows:

ln(ASR)=a+bx+ε;

EAPC=100∗(exp(β)−1).

If the estimated EAPC and the upper boundary of the 95% CI are <0, the ASR can be considered to have a downward trend. Conversely, if the estimated EAPC and the lower boundary of the 95% CI > 0, the ASR can be considered to have an upward trend. Otherwise, the ASR is considered stable. The R program (version 3.6.3) was used for all statistical analyses and graphing the data in this study. *P* values of <0.05 were considered statistically significant.

## RESULTS

### Global decrease from 1990 to 2017 with varied trend in different regions

Globally, the ASIR, ASMR, and ASDR of stomach cancer per 100,000 people decreased from 1990 to 2017—from 21.32 to 15.36, from 19.32 to 10.98, and from 445.86 to 235.94, respectively. Among them, the ASMR (EAPC = −2.2) and the ASDR (EAPC = −2.52) showed the greatest decline, followed by the ASIR (EAPC = −1.36), which agreed with the results of a previous study of GBD 2017 (Table 1) (8).

**Table 1.** Age-standardized incidence rate, ASMR, and age-standardized disability-adjusted life years rate in 1990 and 2017 and their temporal trends from 1990 to 2017

Characteristics	ASIR/incidence rate per 100000(95% UI)						ASMR/death rate per 100000(95% UI)					
	1990			2017			1990			2017		
	Value	Lower	Upper	Value	Lower	Upper	Value	Lower	Upper	Value	Lower	Upper
Overall	21.32	20.9	21.95	15.36	14.97	15.78	19.32	18.91	19.96	10.98	10.77	11.23
Sex												
Male	28.6	27.83	29.34	21.75	21.01	22.59	25.9	25.14	26.59	15.19	14.77	15.67
Female	15.36	14.9	16.1	9.89	9.58	10.2	14.05	13.62	14.81	7.46	7.25	7.68
Age												
15–49 yr	5.03	4.88	5.26	3.49	3.4	3.59	3.63	3.51	3.79	1.95	1.9	2
50–69 yr	59.63	58.28	61.38	41.78	40.42	43.24	51.3	49.99	52.97	26.9	26.28	27.57
70 + years	155.88	152.96	160.23	123.21	120.16	126.85	156.06	153.03	161.15	100.34	98.33	102.65
Sociodemographic index												
Low SDI	13.13	11.89	14.47	8.91	8.39	9.39	13.6	12.35	15.01	9.31	8.74	9.83
Low-middle SDI	12.84	12.14	13.88	9.36	8.94	9.85	13.32	12.6	14.4	9.21	8.84	9.64
Middle SDI	21.45	20.7	22.63	16.44	15.7	17.19	21.81	21.1	23.02	12.37	11.93	12.83
High-middle SDI	27.57	26.87	28.38	21.31	20.25	22.49	27.04	26.37	27.84	14.62	14.02	15.24
High SDI	21.34	21.11	21.58	13.34	12.92	13.81	15.17	15.06	15.3	7.41	7.21	7.63
Region												
High-income Asia-Pacific	57.47	56.5	58.41	29.49	28.18	30.96	32.81	32.44	33.19	14.2	13.66	14.82
Central Asia	23.12	22.62	23.61	14.12	13.52	14.7	23.53	23.03	24.03	14.34	13.76	14.94
East Asia	33.54	32.32	35.53	28.6	27.26	29.98	33.63	32.43	35.68	18.68	17.95	19.48
South Asia	10.35	9.58	11.13	7.15	6.76	7.48	10.68	9.86	11.48	7.45	7.02	7.79
Southeast Asia	12.12	11.17	13.13	6.82	6.37	7.34	12.7	11.73	13.8	7.02	6.57	7.55
Australasia	11.69	11.38	12.03	8.78	7.91	9.68	8.45	8.27	8.63	4.44	4.08	4.83
Caribbean	10.61	10.1	11.2	7.94	7.41	8.55	11.01	10.48	11.62	7.25	6.77	7.81
Central Europe	16.53	16.19	16.83	9.38	9.09	9.67	16.82	16.48	17.12	8.63	8.38	8.88
Eastern Europe	29.33	28.55	30.15	17.74	17.21	18.29	26.55	26.04	26.99	12.77	12.47	13.13
Western Europe	16.74	16.47	17.04	10.49	9.96	10.98	14.06	13.93	14.2	6.43	6.18	6.67
Andean Latin America	24.83	23.73	26	16.6	15.23	17.95	26.69	25.52	27.94	17.11	15.75	18.55
Central Latin America	15.56	15.32	15.87	12.93	12.35	13.54	15.95	15.74	16.17	9.32	8.98	9.66
Southern Latin America	18.76	18.36	19.17	12.35	11.47	13.35	19.9	19.48	20.35	12.29	11.46	13.24
Tropical Latin America	15.8	15.5	16.08	9.45	9.25	9.68	16.8	16.51	17.09	9.25	9.07	9.43
North Africa and Middle East	13.46	12.25	14.56	8.66	8.26	9.08	14.01	12.76	15.16	8.76	8.35	9.17
High-income North America	8.15	8.01	8.31	6.49	6.29	6.71	5.94	5.88	6.01	3.56	3.47	3.66
Oceania	16.18	13.89	18.57	13.83	11.72	16.09	16.38	14.18	18.76	14	11.93	16.15
Central Sub-Saharan Africa	10.85	9.36	12.38	7.13	6.28	7.99	11.49	9.91	13.1	7.59	6.7	8.47
Eastern Sub-Saharan Africa	10.65	9.47	11.76	6.37	5.93	6.84	11.15	9.96	12.25	6.77	6.3	7.26
Southern Sub-Saharan Africa	7.14	6.75	7.6	5.19	4.97	5.44	7.47	7.03	7.98	5.52	5.28	5.78
Western Sub-Saharan Africa	9.72	8.81	10.68	7.72	7.05	8.48	10.45	9.48	11.47	8.41	7.7	9.23

Characteristics	Age-standardized DALYs rate per 100,000(95% UI)						EAPC (95% CI)								
	1990			2017			ASIR/incidence rate(1990–2017)			ASMR/death rate(1990–2017)			ASDR/age-standardized DALYs rate (1990–2017)		
	Value	Lower	Upper	Value	Lower	Upper	Value	Lower	Upper	Value	Lower	Upper	Value	Lower	Upper
Overall	445.86	435.03	461.22	235.94	231.11	241.33	−1.36	−1.47	−1.25	−2.2	−2.29	−2.12	−2.52	−2.61	−2.43
Sex															
Male	582.28	563.91	600.16	317.75	308.75	327.91	−1.16	−1.26	−1.06	−2.07	−2.15	−1.98	−2.38	−2.46	−2.29
Female	326.31	314.56	343.88	163	158.28	167.8	−1.77	−1.88	−1.65	−2.49	−2.58	−2.4	−2.78	−2.9	−2.67
Age															
15–49 yr	174.73	168.72	182.6	91.73	89.56	93.99	−1.74	−1.98	−1.51	−2.75	−2.99	−2.51	−2.84	−3.07	−2.6
50–69 yr	1,448.46	1,411.65	1,495.27	754.15	737.02	772.45	−1.6	−1.82	−1.38	−2.65	−2.8	−2.49	−2.62	−2.76	−2.48
70 + yr	2,168.88	2,124.67	2,242.78	1,294.66	1,265.83	1,326.46	−0.93	−0.99	−0.86	−1.66	−1.74	−1.57	−1.96	−2.07	−1.84
Socio-demographic index															
Low SDI	334.12	301.04	369.07	222.02	209.01	234.67	−1.48	−1.57	−1.39	−1.44	−1.52	−1.35	−1.57	−1.67	−1.46
Low-middle SDI	321.49	303.5	346.89	206.25	197.71	215.94	−1.22	−1.27	−1.18	−1.41	−1.47	−1.36	−1.72	−1.78	−1.66
Middle SDI	500	482.14	527.61	262.8	253.08	272.92	−1.15	−1.29	−1.01	−2.2	−2.36	−2.04	−2.53	−2.67	−2.39
High-middle SDI	634.43	617.88	653.07	311.36	297.76	325.21	−1.11	−1.28	−0.94	−2.41	−2.54	−2.29	−2.86	−3.01	−2.71
High SDI	330.13	326.83	333.49	145.14	141.18	149.32	−1.86	−1.93	−1.8	−2.76	−2.82	−2.7	−3.16	−3.22	−3.1
Region															
High-income Asia Pacific	743.95	733.53	754.31	280	268.57	292.34	−2.53	−2.6	−2.46	−3.22	−3.29	−3.16	−3.7	−3.76	−3.64
Central Asia	593.36	580.65	605.94	339.99	324.95	354.63	−1.76	−1.92	−1.6	−1.76	−1.91	−1.6	−2.02	−2.17	−1.88
East Asia	774.92	747.03	819.99	389.54	373.53	407.16	−0.72	−0.9	−0.54	−2.23	−2.41	−2.05	−2.71	−2.89	−2.54
South Asia	265.99	247.87	285.51	178.16	168.47	186.24	−1.44	−1.57	−1.32	−1.41	−1.52	−1.29	−1.55	−1.69	−1.41
Southeast Asia	296.89	272.63	321.66	154.05	143.46	166.35	−2.35	−2.45	−2.25	−2.4	−2.5	−2.31	−2.61	−2.7	−2.53
Australasia	168.73	165.12	172.44	88.37	80.66	96.63	−1.34	−1.48	−1.21	−2.64	−2.78	−2.49	−2.65	−2.8	−2.49
Caribbean	238.52	225.28	254.04	164.35	152.48	178.7	−1.12	−1.3	−0.94	−1.62	−1.8	−1.44	−1.47	−1.65	−1.28
Central Europe	379.36	370.92	386.49	189.86	184	196.01	−2.06	−2.11	−2.01	−2.47	−2.51	−2.43	−2.57	−2.62	−2.53
Eastern Europe	664.07	648.47	676.34	308.68	300.67	317.32	−2.07	−2.29	−1.84	−2.98	−3.23	−2.73	−3.19	−3.47	−2.9
Western Europe	279.26	276.29	282.33	125.36	120.24	130.38	−1.83	−1.99	−1.68	−3	−3.14	−2.86	−3.05	−3.17	−2.93
Andean Latin America	573.18	545.68	602.03	353.5	322.43	384.45	−1.64	−1.81	−1.48	−1.81	−1.99	−1.63	−1.89	−2.07	−1.71
Central Latin America	333.78	329.25	338.5	203.78	195.82	211.69	−1	−1.11	−0.9	−2.3	−2.4	−2.2	−2.09	−2.18	−2.01
Southern Latin America	425.41	416.11	434.82	253.76	234.69	275.14	−1.46	−1.51	−1.41	−1.69	−1.74	−1.64	−1.8	−1.86	−1.75
Tropical Latin America	363.49	356.82	370.07	203.03	199.32	207.13	−1.99	−2.09	−1.89	−2.3	−2.42	−2.19	−2.23	−2.35	−2.11
North Africa and Middle East	327.16	295.27	355.96	189.03	178.95	198.92	−1.62	−1.71	−1.54	−1.71	−1.78	−1.64	−2.04	−2.11	−1.98
High-income North America	122.6	121.13	124.2	74.54	72.29	76.71	−1.19	−1.33	−1.06	−2.1	−2.22	−1.99	−2.08	−2.2	−1.95
Oceania	423.76	356.52	492.24	358.42	297.25	423.57	−0.47	−0.52	−0.41	−0.48	−0.53	−0.43	−0.47	−0.54	−0.39
Central Sub-Saharan Africa	265.61	228.05	304.97	172.96	151.34	195	−1.63	−1.71	−1.56	−1.61	−1.68	−1.53	−1.69	−1.77	−1.6
Eastern Sub-Saharan Africa	268.33	235.83	300.16	155.81	145.03	167.4	−2.2	−2.33	−2.06	−2.13	−2.26	−2	−2.33	−2.48	−2.19
Southern Sub-Saharan Africa	177.79	167.24	189.76	123.25	117.33	129.63	−1.29	−1.88	−0.7	−1.23	−1.81	−0.65	−1.47	−2.12	−0.82
Western Sub-Saharan Africa	220.28	198.89	242.75	167.93	152.95	185.7	−0.76	−0.85	−0.67	−0.71	−0.8	−0.62	−0.93	−1.03	−0.83

ASMR, age-standardized mortality rate; SDI, sociodemographic index.

**Figure 1.**
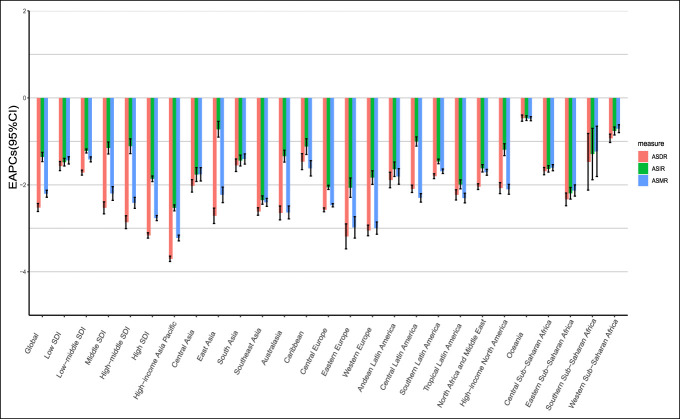
EAPC in the ASR of stomach cancer at the regional level from 1990 to 2017. EAPC, estimated annual percentage change; ASR, age-standardized rate.

From 1990 to 2017, the ASMR and ASDR of stomach cancer was significantly reduced in most areas of Asia, especially in the high-income Asia-Pacific region, which showed the fastest decline in the ASMR and ASDR (EAPC = −3.22 and EAPC = −3.70, respectively). Furthermore, there was a significant decline of the ASMR and ASDR in Europe. For instance, Eastern Europe (EAPC [ASMR] = −2.98 and EAPC [ASDR] = −3.16) and Western Europe (EAPC [ASMR] = −3 and EAPC [ASDR] = −3.17) have made obvious progress in controlling the mortality and DALY rates of stomach cancer. The fastest decline in the ASIR was also observed in the high-income Asia-Pacific region (EAPC = −2.53), followed by Southeast Asia (EAPC = −2.35) (Figure 1, Table 1).

The absolute value of EAPC (EAPC [ASIR] = −0.47, EAPC [ASMR] = −0.48, and EAPC [ASDR] = −0.47) in the Oceania region (negative for all regions) was the lowest, far lower than the global average value. In addition, the ASMR and ASDR decreased in the East Asia (EAPC = −2.23 and EAPC = −2.71, respectively), which was larger than the global average value, but the decrease in the ASIR in the East Asia (EAPC = −0.72) was very slow (Figure 1, Table 1).

### Variation in the trend of stomach cancer burden in 195 countries

**Figure 2.**
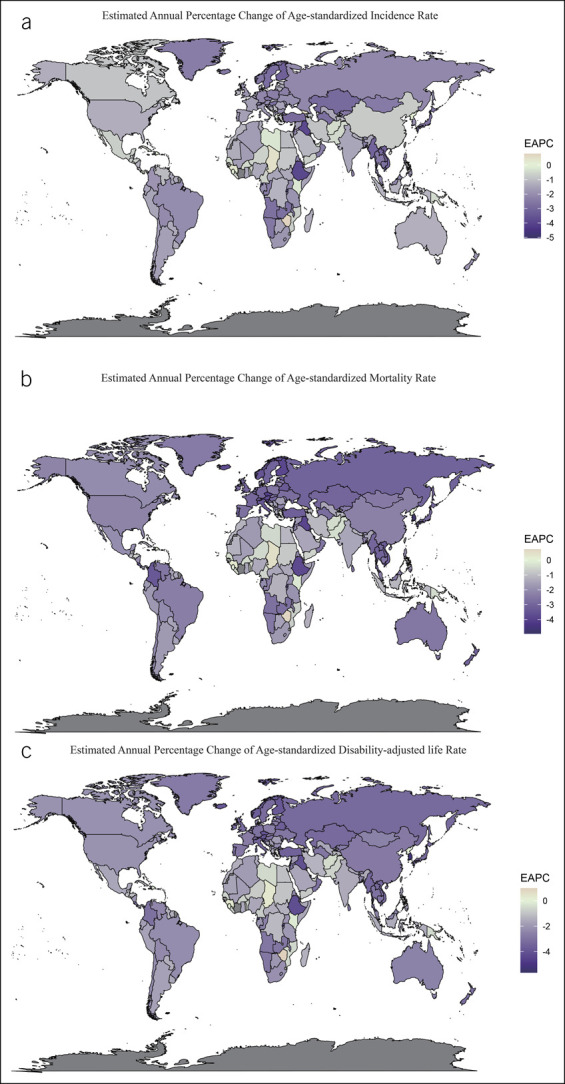
EAPC in the ASR of stomach cancer in 195 countries and territories from 1990 to 2017. (**a**) The EAPC in the ASIR of stomach cancer from 1990 to 2017. (**b**) The EAPC in the ASMR of stomach cancer from 1990 to 2017. (**c**) The EAPC in the ASDR of stomach cancer from 1990 to 2017. ASIR, age-standardized incidence rate; ASMR, age-standardized mortality rate; ASDR, age-standardized disability-adjusted life years rate; EAPC, estimated annual percentage change; ASR, age-standardized rate.

The ASIR, ASMR, and ASDR of stomach cancer in most countries showed a significant downward trend from 1990 to 2017. However, the variation in the trends of the stomach cancer burden in different regions was inconsistent, and the variation in the trends of the ASMR, ASDR, and ASIR in the same region was also different (Figure 2).

The changing trends of stomach cancer burden varied greatly across countries. Although the ASIR, ASMR, and ASDR in Equatorial Guinea (EAPC = −5.05, EAPC = −4.93, and EAPC = −5.45, respectively) decreased, these rates in Zimbabwe (EAPC = 0.8, EAPC = 0.75, and EAPC = 0.99, respectively) increased. The EAPC of the ASIR was positive in 10 countries (Dominican, Sierra Leone, North Korea, Sao Tome and Principe, Lesotho, Guinea, El Salvador, Republic Chad, Georgia, Cyprus, and Zimbabwe), and most of them are mostly in Africa (Figure 2; see Supplementary Table 1, Supplementary Digital Content 1, http://links.lww.com/CTG/A691).

Some countries with a high baseline ASR in 1990 also showed a significant downward trend from 1990 to 2017. For example, in South Korea, the ASR of stomach cancer (ASIR = 49.24; ASMR = 47.08; ASDR = 1,185.41) was the fifth highest in 1990, among which the ASMR and ASDR (EAPC = −4.88 and EAPC = −5.25, respectively) showed the greatest decrease. By contrast, the ASIR in South Korea (EAPC = −1.97) decreased slowly. Similar features can be found in other countries, such as Singapore, Czech Republic, and Estonia. In countries with a high SDI value and a low baseline ASR in 1990, mostly European countries, the ASR decline was higher (Figure 2, see Supplemental Table 1, Supplementary Digital Content 1, http://links.lww.com/CTG/A691).

### Age-specific burden of stomach cancer and EAPC

**Figure 3.**
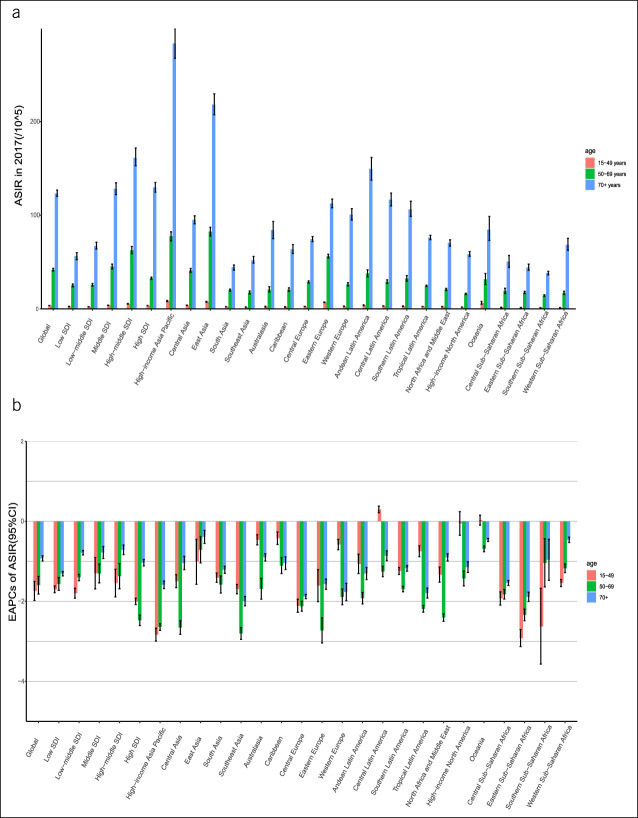
ASIR and EAPC of stomach cancer in all age groups at the regional level. (**a**) The ASIR in all age groups in 2017. (**b**) The EAPC in the ASIR in all age groups from 1990 to 2017. ASIR, age-standardized incidence rate; EAPC, estimated annual percentage change; SDI, sociodemographic index.

The population was divided into the following 3 age groups: 15–49 years, 50–69 years, and 70 years or older. The incidence of stomach cancer per 100,000 people in persons younger than 15 years was 0; therefore, they were not included in this study. In 2017, people aged 70 years or older had the most serious burden of stomach cancer (ASIR = 123.20; ASMR = 100.34; and ASDR = 1,294.66) (Figure 3; see Supplemental Figure 1, Supplementary Digital Content 1, http://links.lww.com/CTG/A691). From 1990 to 2017, the ASIR, ASMR, and ASDR of most age groups in the 21 GBD regions showed downward trends, but the ASIR of stomach cancer in people aged 15–49 years in Central and Latin America showed an upward trend (EAPC = 0.30) (Figure 3b). The stomach cancer burden decreased most slowly for population aged 70 years or older in most regions. In particular, in East Asia, which had the highest burden of stomach cancer in the population of 70 years or older, the decreases in the ASIR, ASMR, and ASDR were lower than those of the global average values (EAPC = −0.39, EAPC = −0.97, and EAPC = −1.94, respectively) (Figure 3; see Supplemental Figure 1, Supplementary Digital Content 1, http://links.lww.com/CTG/A691).

### Sex-specific burden of stomach cancer and EAPC

**Figure 4.**
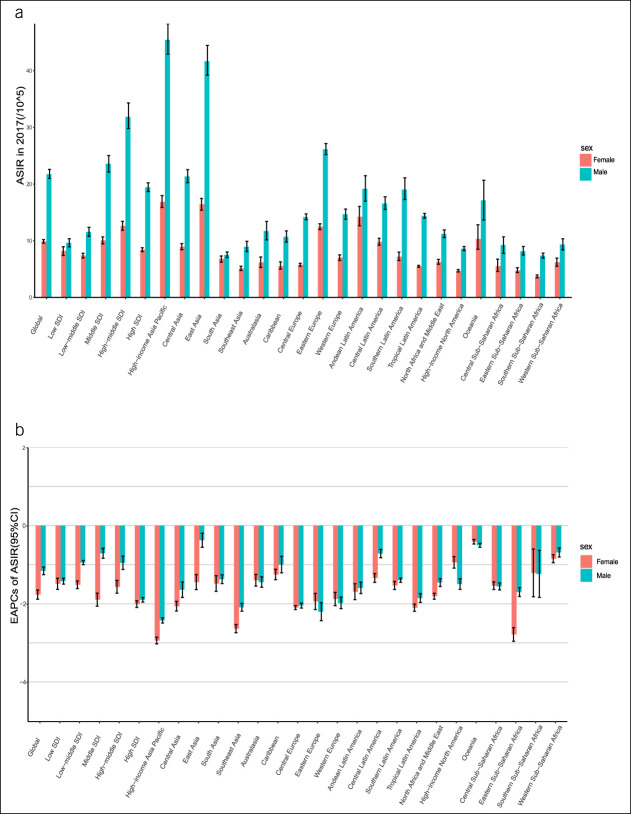
ASIR and EAPC of stomach cancer according to sex at the regional level. (**a**) The ASIR according to sex in 2017. (**b**) The EAPC in the ASIR according to sex from 1990 to 2017. ASIR, age-standardized incidence rate; EAPC, estimated annual percentage change; SDI, sociodemographic index.

In 2017, the ASIR, ASMR, and ASDR of stomach cancer (per 100,000) in male patients worldwide (ASIR = 21.74; ASMR = 15.19; and ASDR = 317.75) were much higher than those in female patients (ASIR = 9.89; ASMR = 7.46; and ASDR = 163.00) (Figure 4, see Supplemental Figure S, Supplementary Digital Content 1, http://links.lww.com/CTG/A691), which was the most apparent in Asia. The decrease in the ASIR, ASMR, and ASDR of stomach cancer in female patients worldwide from 1990 to 2017 (EAPC = −1.77, EAPC = −2.49, and EAPC = −2.78, respectively) was sharper than that in male patients (EAPC = −1.15, EAPC = −2.07, and EAPC = −2.38, respectively). The stomach cancer burden in female patients decreased faster in most regions, but in parts of Europe and Africa, the opposite trend was observed (Figure 4; see Supplemental Figure 2, Supplementary Digital Content 1, http://links.lww.com/CTG/A691).

### SDI negatively correlated with mortality, life span, and their annual changes

**Figure 5.**
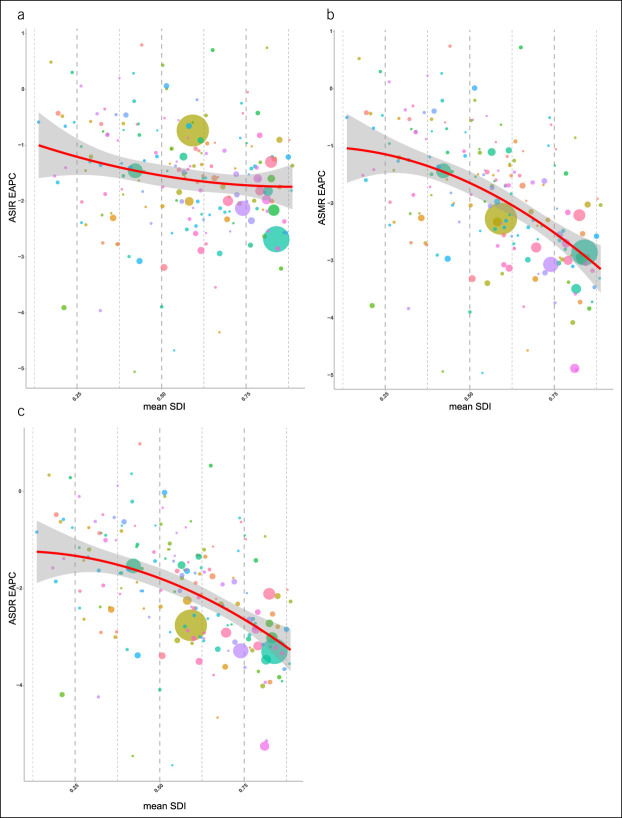
Correlation between EAPC and mean SDI from 1990 to 2017. (**a**) Correlation between the EAPC of the ASIR and the mean SDI from 1990 to 2017 (*r* = −0.18, *P* < 0.05). (**b**) Correlation between the EAPC of the ASMR and the mean SDI from 1990 to 2017 (*r* = −0.50, *P* < 0.01). (**c**) Correlation between the EAPC of the ASDR and the mean SDI from 1990 to 2017 (*r* = −0.46, *P* < 0.01). The circles represent the 195 countries and territories. The size of the circle represents the current number of cases. The *γ* indices and *P* values are derived from the Pearson correlation analysis. ASIR, age-standardized incidence rate; ASMR, age-standardized mortality rate; ASDR, age-standardized disability-adjusted life years rate; EAPC, estimated annual percentage change; SDI, sociodemographic index.

Correlation analyses were conducted between SDI values of 195 countries and the ASIR, ASMR, and ASDR of stomach cancer in 2017. In addition, correlation analyses were conducted between SDI values of 195 countries and the EAPC of the ASRs from 1990 to 2017. The results showed that SDI had a significant negative correlation with ASMR (*P* < 0.01, r = −0.28) and ASDR (*P* < 0.01, r = −0.31) in 2017. However, there was no significant correlation between SDI and ASIR (*P* = 0.88) (see Supplemental Figure 3, Supplementary Digital Content 1, http://links.lww.com/CTG/A691). Because SDI values changed annually in each country from 1990 to 2017, we calculated the mean SDI in each country for these years and analyzed its correlation with the EAPC values of the ASIR, ASMR, and ASDR. The mean SDI had a significant negative correlation with EAPCs in the ASIR (*P* < 0.05, r = −0.18), ASMR (*P* < 0.01, r = −0.50), and ASDR (*P* < 0.01, r = −0.46) (Figure 5; see Supplemental Figure 3, Supplementary Digital Content 1, http://links.lww.com/CTG/A691). Hence, decreases in the ASIR, ASMR, and ASDR of stomach cancer were greater in countries with higher SDI values.

## DISCUSSION

Globally, stomach cancer is an important threat to human health. Stomach cancer had the fifth and third highest incidence and mortality rates, respectively, among all malignant tumors globally in 2018 (1) and the total number of cases and deaths has increased from 1990 to 2017. This study systematically analyzed the EAPC of the ASIR, ASMR, and ASDR of stomach cancer in 195 countries and regions from 1990 to 2017. The decline in stomach cancer burden was lower in male patients and the 70 years or older group, and there was a significant negative correlation between SDI and the ASMR, ASDR, and EAPC.

The ASIR, ASMR, and ASDR of stomach cancer showed a downward trend from 1990 to 2017, but decreases in the ASMR and ASDR were greater than those in the ASIR, and the decreasing rate of the stomach cancer burden varied across different regions, consistent with the findings of a previous study of the GBD 2020 (8). With the improvements in sanitation, changes in diet (increases in the consumption of fruit and vegetables and decreases in the consumption of salt), widespread antibiotic use, and predominantly the decreasing prevalence of *H. pylori* infection, the incidence and mortality rates of stomach cancer are declining. Some countries are taking active measures to reduce the mortality rate of stomach cancer. For example, in high-income Asia-Pacific region countries, such as South Korea and Japan, national screening programs for stomach cancer have been implemented. Singapore has also started a research consortium named the Singapore Stomach Cancer Consortium to help the early discovery of stomach cancer (13). Early screening programs can effectively reduce the mortality rate of stomach cancer. However, these programs can yield a higher rate of diagnosis of stomach cancer.

We found that the stomach cancer burden in male patients is a public health problem that deserves attention. In 2017, the ASIR, ASMR, and ASDR of stomach cancer in male patients were almost twice higher than those in female patients. Furthermore, the ASIR, ASMR, and ASDR of stomach cancer in female patients decreased more than those in male patients from 1990 to 2017, but this varied across different regions. The incidence of stomach cancer in male patients was high, especially in the high-income Asia-Pacific region, East Asia, and Eastern Europe. The main reason for this may be that male patients had higher smoking rates than female patients in most regions. Implementation of different smoking cessation strategies can help further reduce the incidence of stomach cancer in male patients. In addition, the differences in other risk factors between male and female patients should be considered, such as high-sodium diet (especially in East Asia), drinking habits, occupational exposure, hormone levels, and drug-use habits (2,14,15).

The stomach cancer burden is also closely related to age. We found that people aged 70 years or older had the highest ASIR, ASMR, and ASDR of stomach cancer, worldwide, especially in the high-income Asia-Pacific and East Asia regions. The decreases in the ASIR, ASMR, and ASDR were the highest among people aged 15–49 years, followed by those aged 50–69 years; they were the lowest among people aged 70 years or older. The proportion of the older population will increase in all regions between 2019 and 2050, and the largest increase (+312 million persons) is projected to occur in Eastern and South-Eastern Asia (16), and this will yield more cases of stomach cancer (13,14). The resulting burden of stomach cancer cannot be ignored.

In 2017, in a cross-sectional view, countries with higher SDI values had the lowest ASMR and ASDR for stomach cancer. Moreover, from 1990 to 2017, in countries with higher mean SDI values, the ASIR, ASMR, and ASDR of stomach cancer decreased faster. Thus, in some countries with high SDI values and low baseline ASRs in 1990, the decline in the ASR was higher. In countries with higher SDI values, the available treatment options, diagnosis and treatment model, and accessibility and quality of health care could be improved to reduce the mortality and DALY rates of stomach cancer and, thus, the stomach cancer burden (17,18). In countries with high mean SDI values, the condition of food preservation was better with refrigeration, and the intake of fresh vegetables, which has a protective effect on the occurrence and development of stomach cancer, was higher (22). Moreover, in countries and regions with higher SDI values, such as Taiwan (province of China), South Korea, Japan, and some European countries, more resources could be invested for the early diagnosis of stomach cancer and the screening and eradication of *H. pylori.* (8,18,20) Significant declines in the ASIR, ASMR, and ASDR of stomach cancer were observed in all these areas. However, a higher level of early diagnosis of stomach cancer can offset the larger decline in ASIR in countries with higher SDI values. This could also explain why the relevance between the EAPC of the ASIR and the mean SDI is weak.

Tobacco is a confirmed risk factor of stomach cancer (21). Significant declines in prevalence of smoking were observed in countries with high SDI values, and all-cause DALY attributable to smoking in male patients decreased by 11.8% in countries with high SDI values from 2005 to 2015 (20). The decline in tobacco use can help decrease the stomach cancer burden in countries with high SDI values. Moreover, the mortality attributable to tobacco use was higher in regions with low and low-middle SDI values (21). Hence, implementation of tobacco cessation strategies is very important for the prevention of stomach cancer in all countries. Indonesia has very high levels of smoking. The mean SDI value of Brazil (0.58) was similar to those of Indonesia (0.56) . By implementing tobacco control policies, Brazil achieved the third largest significant decline in age-standardized smoking prevalence since 1990, with the faster decline in the ASIR for stomach cancer (EAPC = −2.00) than that in Indonesia (EAPC = −1.20).

*H. pylori* is a confirmed risk factor of noncardiac cancer, and its relative risk is as high as 6 (23). Effective policies for screening and eradication of stomach cancer should be considered. According to the Taipei global consensus (20), countries or populations with an incidence of stomach cancer >20 per 100,000 person-years, 10–20 per 100,000 person-years, and <10 per 100,000 person-years are defined as high-risk, intermediate-risk, and low-risk countries or populations, respectively. For high-risk and intermediate-risk populations, screening for young individuals was correlated with higher effectiveness but a lower cost. For the low-risk populations, more cost will show more effectiveness (20). An investment in the screening and eradication of *H. pylori* is effective for stomach cancer prevention in countries that can afford to increase in cost. In regions with low SDI values, it is imperative to increase the accessibility to high level medical technology, supply of medical resources, and modalities of early diagnosis of stomach cancer to reduce the mortality of stomach cancer and alleviate the burden caused by stomach cancer.

This study also has some limitations. First, although we discussed the correlation between SDI and the ASR of stomach cancer, the specific factors affecting the stomach cancer burden in different regions were not studied. Second, stomach cancer can be anatomically divided into cardiac cancer and noncardiac cancer, but this study ignored the different epidemiological risk factors and clinicopathological features between the 2 topographical categories and reported on gastric cancers as a single entity (24) (19). Finally, there are limitations in the quality of the raw data from the GBD. Time lags in available data and absence of data from specific regions, age groups, or periods are unavoidable in the process of data collection, especially in countries with low SDI values, meaning that estimations have to rely more on modelling process (25,26). GBD results are a combination of raw data and estimation correction through various models, including a linear step mixed-effects model and spatiotemporal Gaussian process regression (25,26). Therefore, the source of the raw data should be fully considered when interpreting our results.

In summary, the global ASIR, ASMR, and ASDR at all ages showed a downward trend since 1990, but the incidence of stomach cancer is a major public health problem in some high-risk areas. In the future, more attention should be paid to disease prevention and early diagnosis of stomach cancer. Targeted prevention strategies for stomach cancer should be adopted in different countries and regions in the future. Screening and treating *H. pylori* infection for young adults in high-risk population of stomach cancer, increasing the level of diagnosis and treatment of stomach cancer in countries with low SDI values, and strengthening the implementation of tobacco control policies in all countries could help reduce the burden of stomach cancer. In addition, health education on stomach cancer should be strengthened, and people should be encouraged to eat more fruits and vegetables, give up smoking, and control their daily salt intake as increasing public awareness about stomach cancer is important for its prevention.

## Supplementary Material

SUPPLEMENTARY MATERIAL
