# Supplementary material for: Global Burden and Trends in Incidence, Mortality, and Disability of Stomach Cancer From 1990 to 2017
Source: Clin Transl Gastroenterol. 2021 Oct 5;12(10):e00406. doi: 10.14309/ctg.0000000000000406 (PMC8500568; doi:10.14309/ctg.0000000000000406)
Supplement: SUPPLEMENTARY MATERIAL [file ct9-12-e00406-s001.pdf]

a.

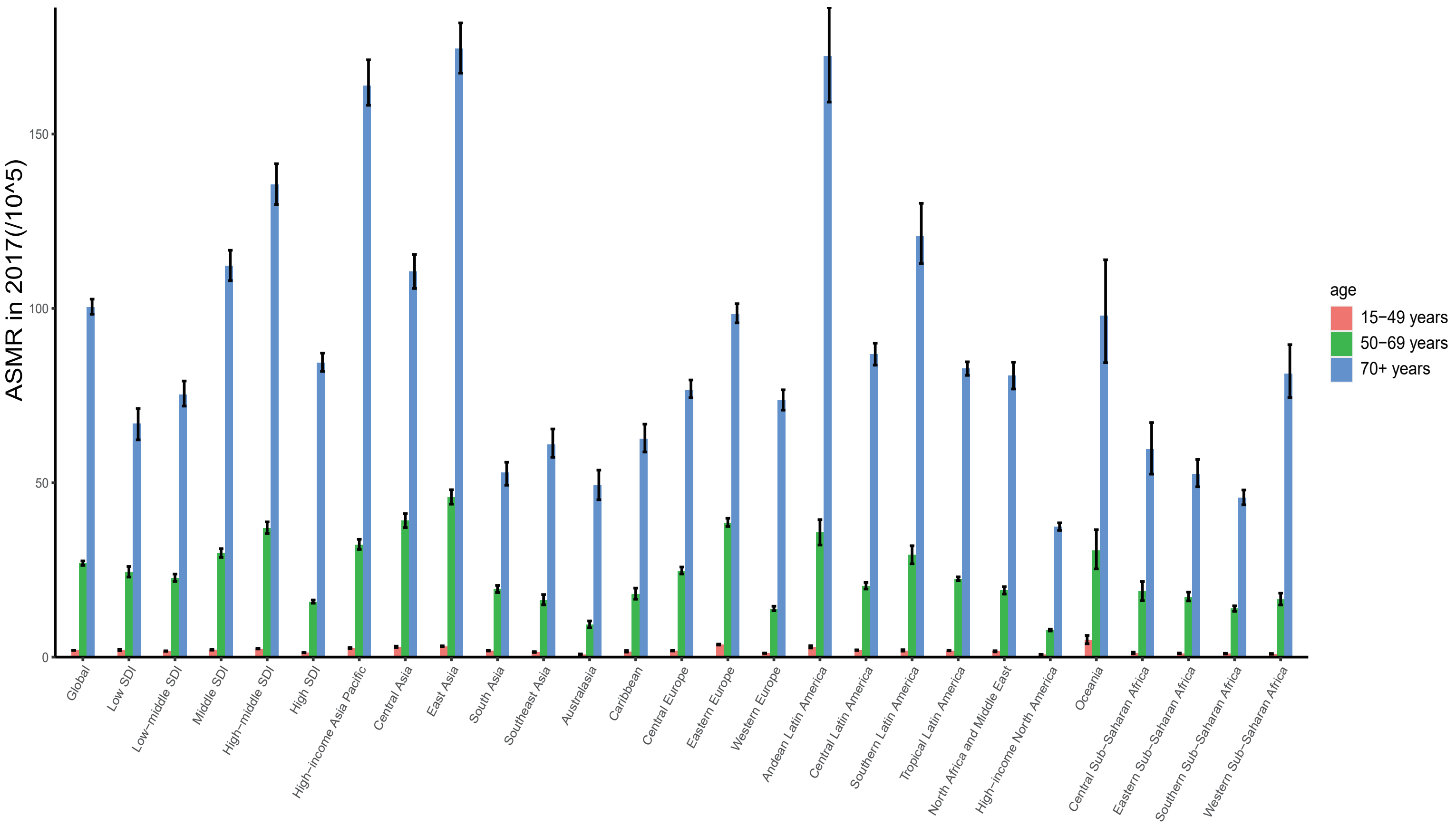

b.

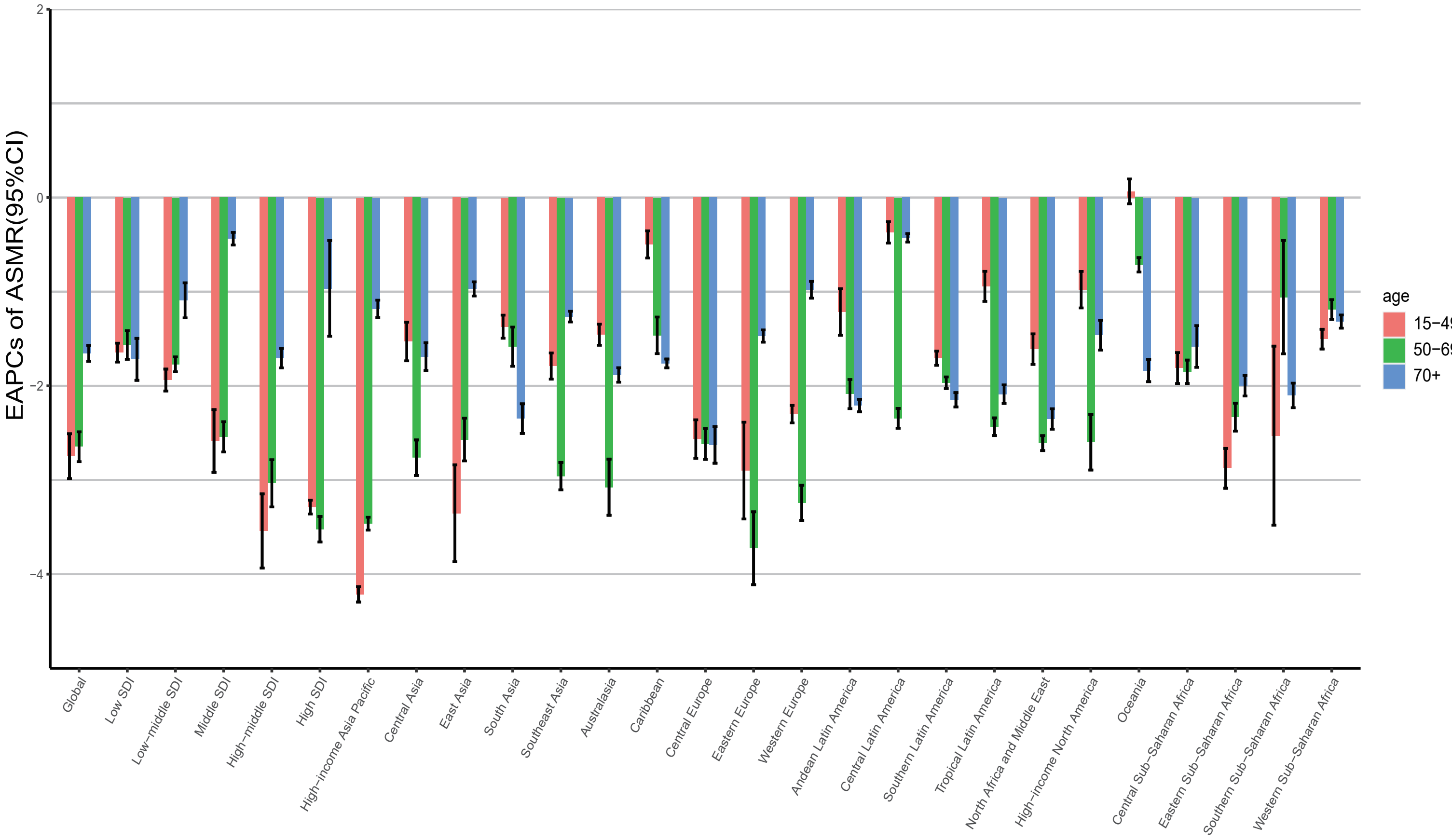

Figure. S1 （Related to Figure 3）The ASMR, ASDR and EAPCs of stomach cancer in all age groups at the regional levels.

a.

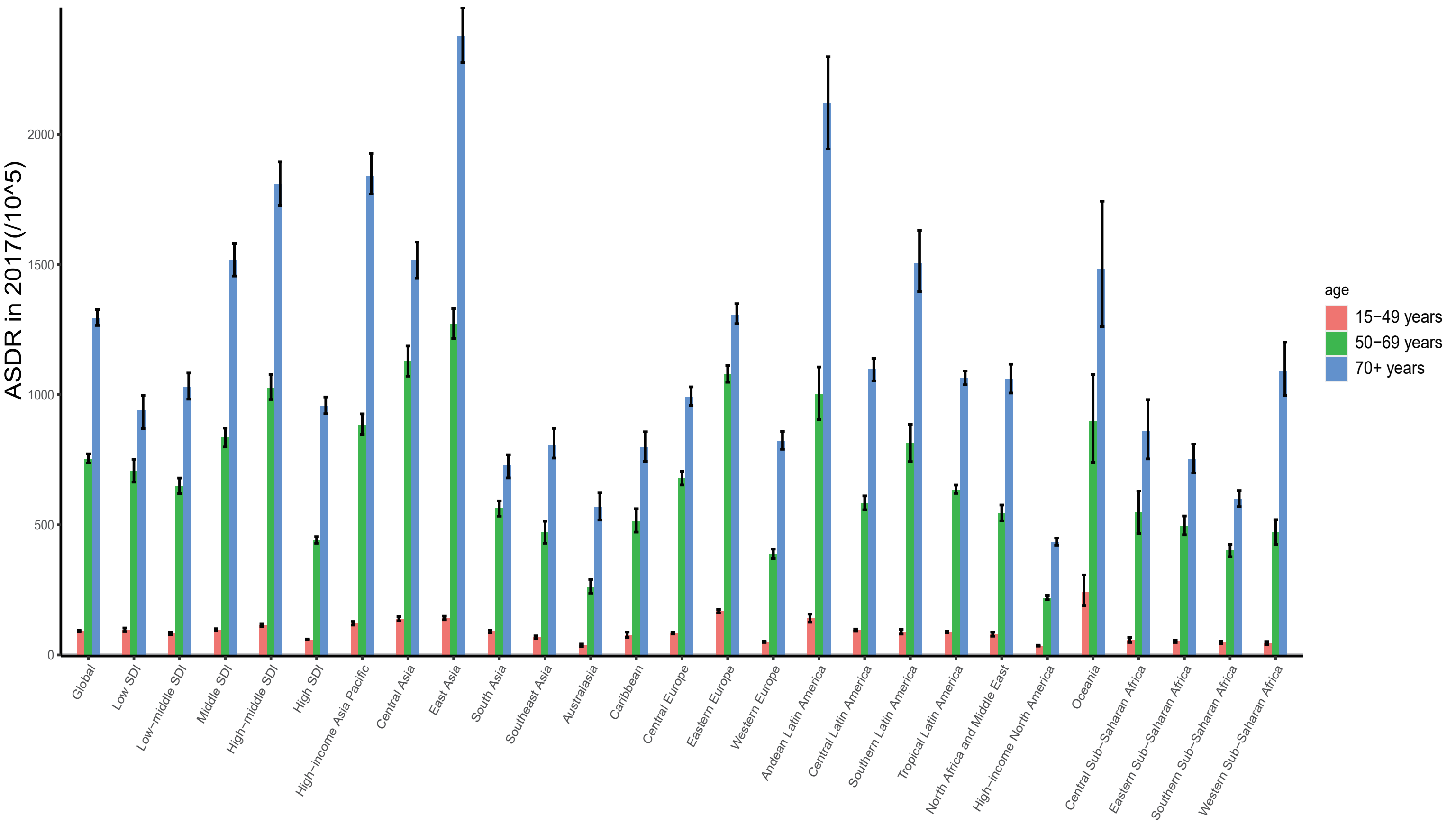

b.

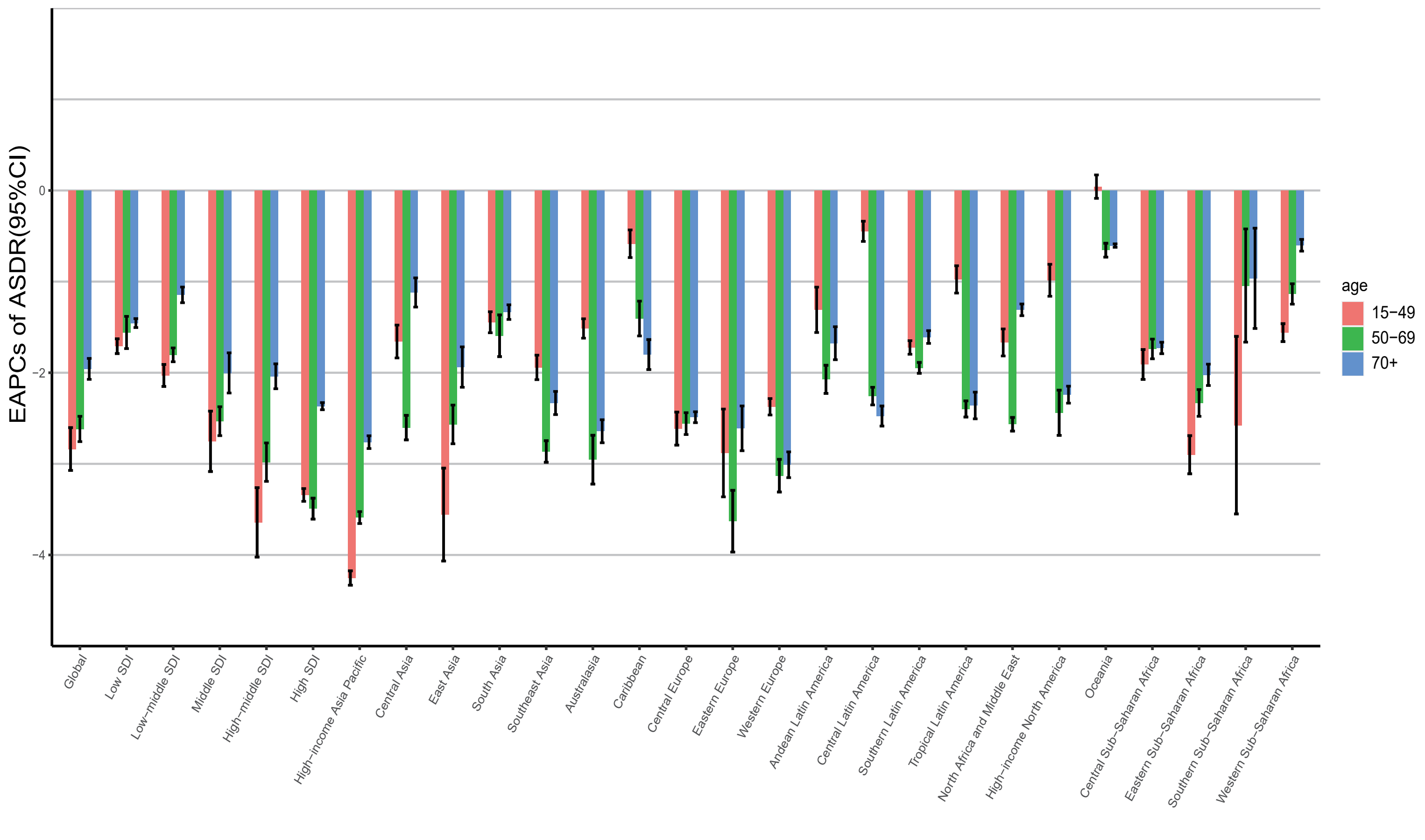

Figure. S1 （Related to Figure 3）The ASMR, ASDR and EAPCs of stomach cancer in all age groups at the regional levels.

a.

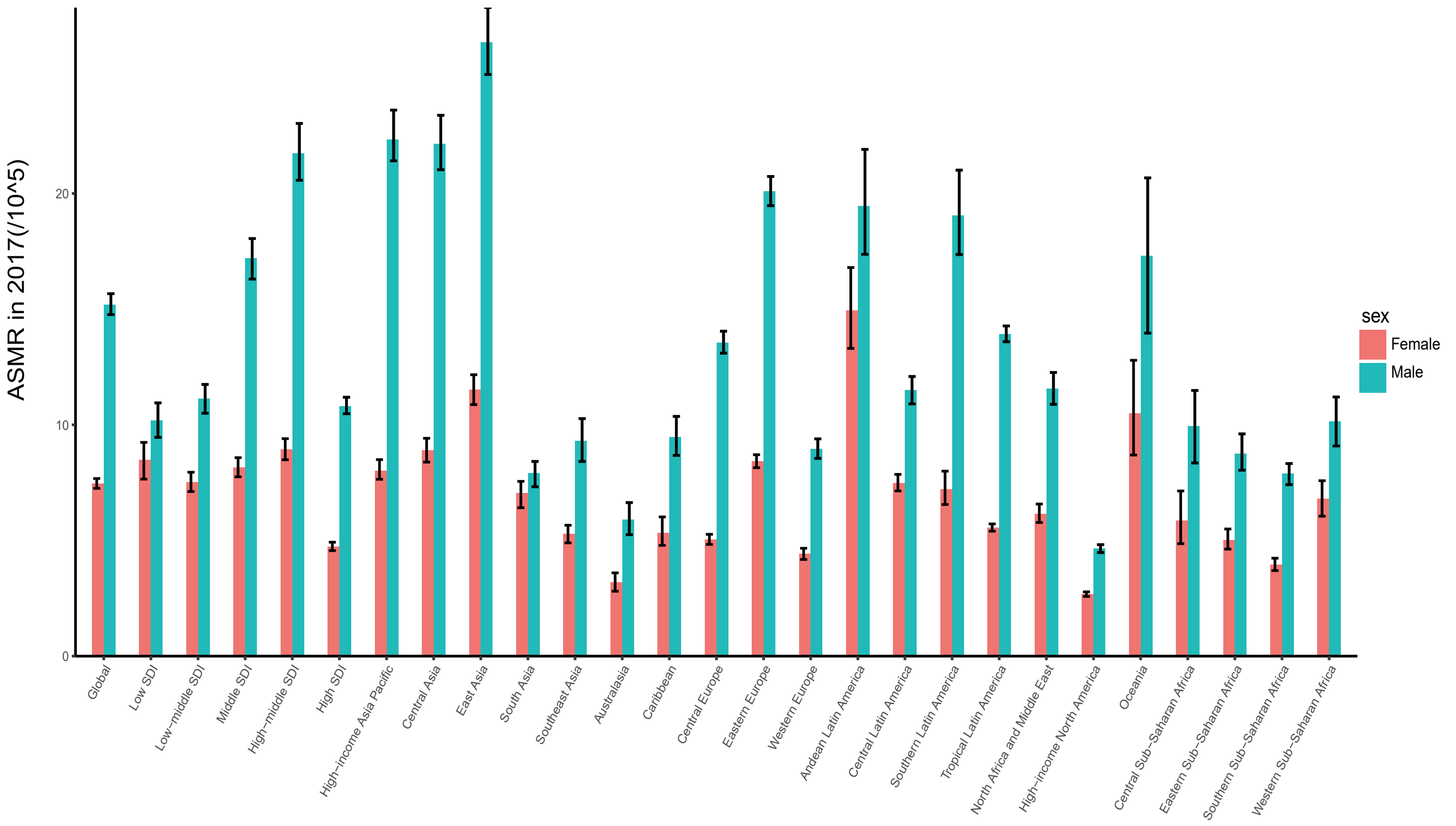

b.

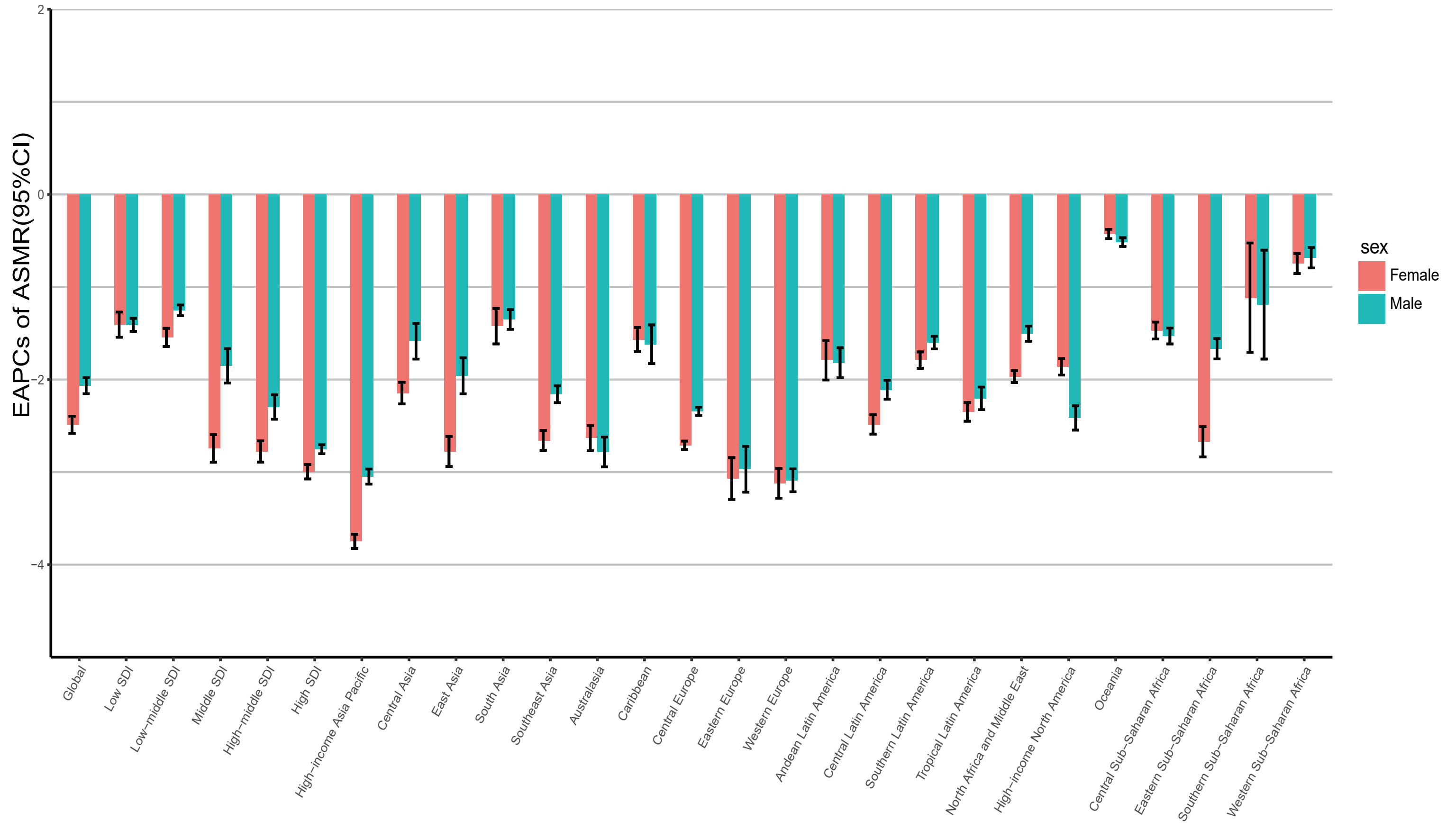

Figure. S2 (Related to Figure 4) The ASMR, ASDR and EAPCs of stomach cancer by gender at the regional levels.

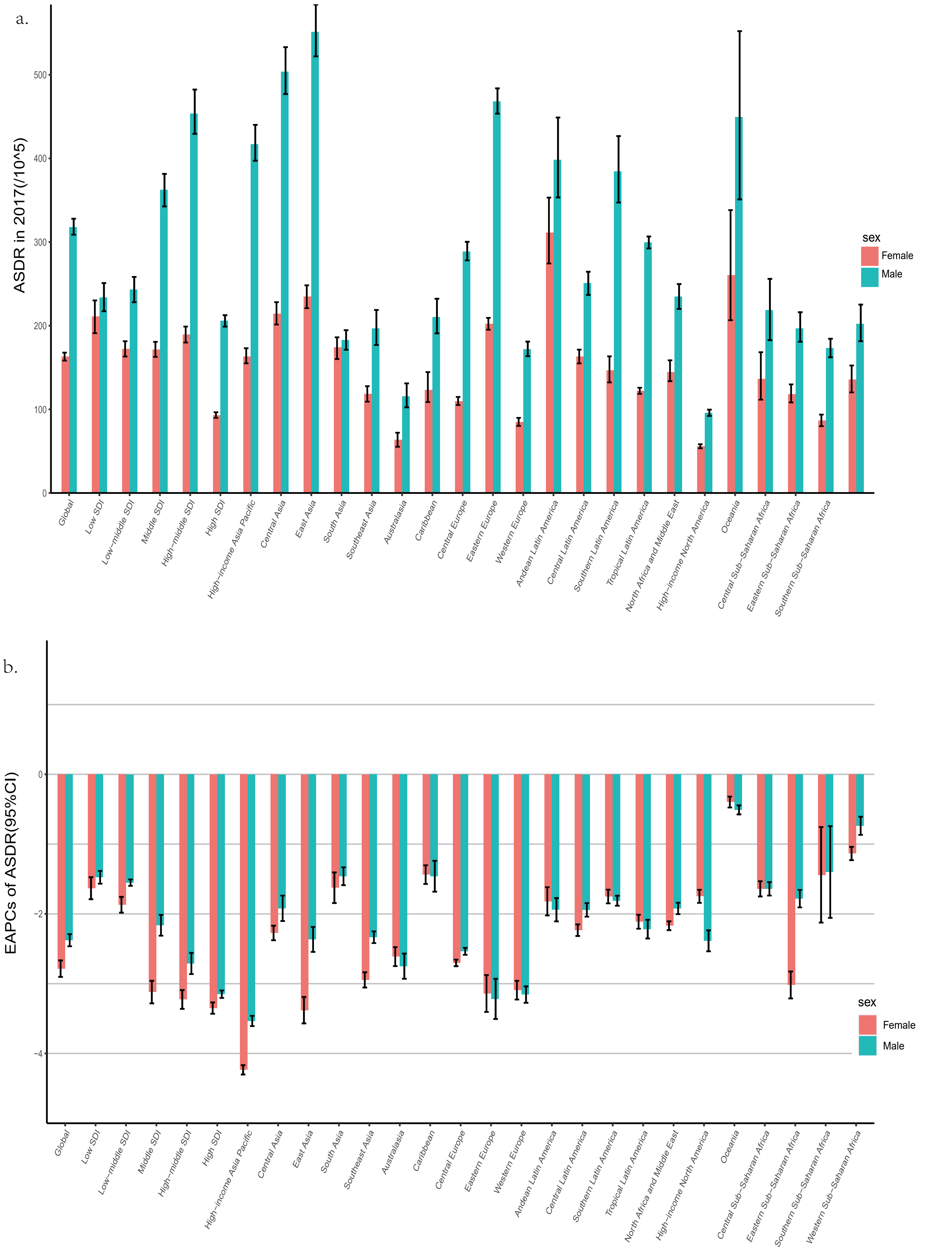

Figure. S2 (Related to Figure 4) The ASMR, ASDR and EAPCs of stomach cancer by gender at the regional levels.

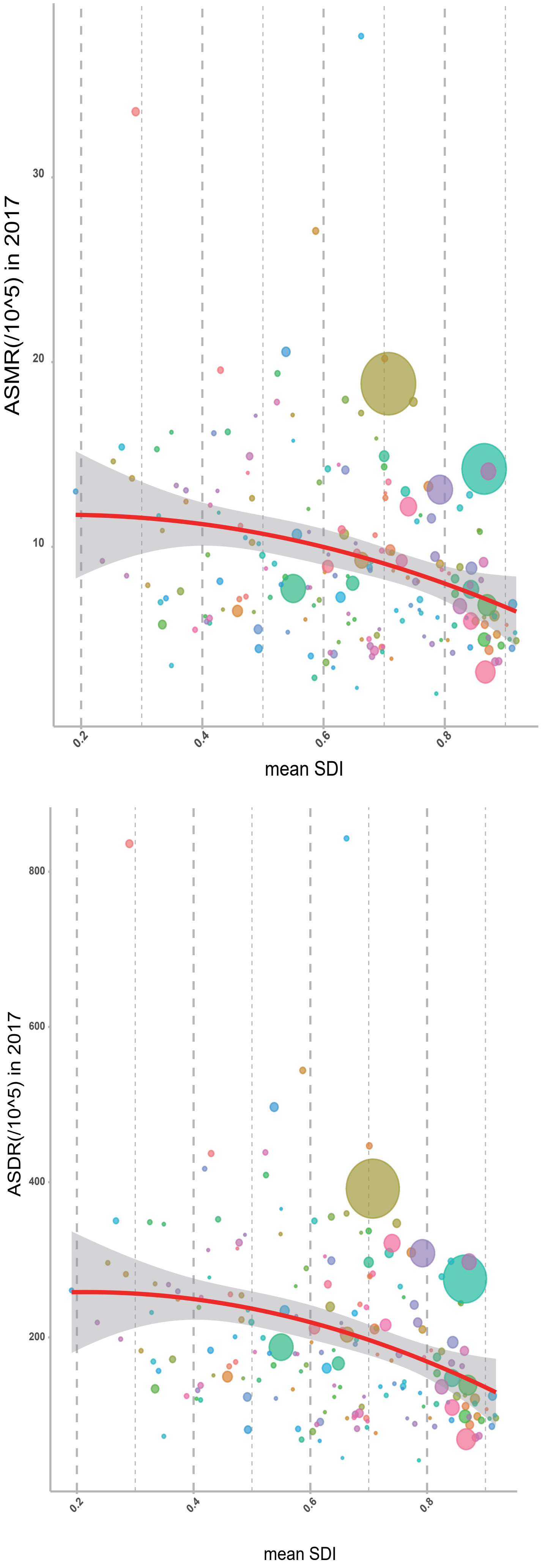

Figure. S3 (Related to Figure 5) The correlation between both ASMR and ASDR and SDI.

	ASIR/Incidence Rate per 100000		ASMR/Death Rate per 100000		ASDR/DALYs Rate per 100000			EAPC		SDI	
	ASIR in 2017	ASIR in 2017	ASMR in 1990	ASMR in 2017	ASDR in 2017	ASDR in 2017	ASIR	ASMR	ASDR	2017 SDI	sdi_mean
Afghanistan	36.36	32.80	36.92	33.56	933.41	836.08	-0.43	-0.42	-0.48	0.29	0.19
Albania	13.65	10.48	14.07	10.63	339.90	242.61	-1.10	-1.18	-1.31	0.69	0.61
Algeria	7.13	4.34	7.58	4.56	168.32	95.84	-1.66	-1.69	-1.88	0.70	0.61
American Samoa	15.62	12.57	16.58	12.90	361.06	278.94	-0.95	-1.09	-1.13	0.70	0.66
Andorra	11.24	9.16	9.38	5.78	179.14	107.54	-0.90	-1.92	-2.03	0.90	0.88
Angola	13.35	6.74	14.01	7.17	334.27	162.74	-2.75	-2.68	-2.90	0.46	0.33
Antigua and Barbuda	13.78	9.23	14.37	8.71	313.37	177.92	-1.57	-1.95	-2.21	0.72	0.67
Argentina	15.04	9.60	15.94	9.83	345.12	210.84	-1.59	-1.69	-1.75	0.71	0.66
Armenia	19.99	12.49	20.30	12.65	513.01	278.81	-1.55	-1.51	-2.12	0.70	0.62
Australia	11.65	8.96	8.16	4.42	161.86	87.45	-1.25	-2.53	-2.53	0.87	0.83
Austria	17.26	10.20	15.31	5.80	309.80	111.38	-2.00	-3.72	-3.93	0.87	0.83
Azerbaijan	23.05	19.51	23.63	20.19	585.45	446.79	-0.97	-0.92	-1.34	0.70	0.63
Bahamas	12.22	8.35	12.59	8.12	290.22	181.49	-1.41	-1.68	-1.78	0.76	0.72
Bahrain	10.29	3.85	11.22	3.95	223.06	76.76	-4.35	-4.56	-4.66	0.71	0.67
Bangladesh	12.37	6.17	12.93	6.53	313.80	149.44	-2.30	-2.25	-2.43	0.46	0.36
Barbados	14.50	8.92	15.26	8.34	314.28	170.26	-1.92	-2.39	-2.42	0.74	0.70
Belarus	31.99	18.57	30.31	13.27	770.17	309.43	-2.32	-3.32	-3.61	0.77	0.69
Belgium	13.05	7.74	12.25	5.26	221.60	98.30	-1.74	-2.92	-2.79	0.89	0.85
Belize	11.24	8.36	12.12	8.63	250.86	193.75	-1.33	-1.49	-1.19	0.60	0.52
Benin	14.45	11.46	15.50	12.45	327.43	249.61	-0.57	-0.52	-0.74	0.37	0.29
Bermuda	11.01	7.72	11.34	4.95	235.37	96.08	-1.02	-2.94	-3.22	0.81	0.77
Bhutan	10.02	5.69	10.44	6.07	253.96	133.09	-2.10	-2.00	-2.41	0.57	0.45
Bolivia	42.04	24.90	45.13	27.10	991.37	544.02	-2.09	-2.03	-2.37	0.59	0.50
Bosnia and Herzegovina	12.21	9.33	12.72	9.69	299.75	211.80	-1.34	-1.34	-1.70	0.71	0.62
Botswana	7.68	4.51	8.33	4.99	180.53	95.85	-1.57	-1.51	-1.99	0.66	0.57
Brazil	15.93	9.48	16.94	9.27	366.57	203.58	-2.00	-2.32	-2.25	0.66	0.58
Brunei	23.64	13.65	22.80	10.90	510.89	244.34	-2.15	-2.91	-2.89	0.86	0.80
Bulgaria	16.85	9.54	17.37	9.08	397.72	210.26	-2.04	-2.29	-2.32	0.79	0.73
Burkina Faso	15.68	12.82	16.60	13.71	355.08	281.46	-0.48	-0.43	-0.62	0.28	0.21
Burundi	13.07	7.40	13.73	7.92	338.45	182.80	-2.42	-2.33	-2.63	0.31	0.28
Cambodia	19.41	9.60	20.29	10.24	480.27	222.82	-2.77	-2.69	-3.00	0.48	0.37
Cameroon	14.62	11.58	15.66	12.63	335.30	254.24	-0.61	-0.53	-0.81	0.48	0.39
Canada	12.93	10.50	10.30	6.28	204.66	120.69	-0.90	-2.02	-2.15	0.88	0.85
Cape Verde	24.41	15.60	26.49	17.14	556.21	333.14	-1.63	-1.58	-1.87	0.55	0.43
Central African Republic	14.81	10.36	15.46	10.88	379.90	269.07	-1.45	-1.42	-1.42	0.33	0.28
Chad	13.06	13.46	14.06	14.62	298.01	296.05	0.49	0.53	0.34	0.25	0.17
Chile	30.65	18.59	32.71	17.85	677.72	347.03	-1.74	-2.14	-2.32	0.75	0.68

Table. S1 (Related to Table 1) The age-standardized incidence rate (ASIR), age-standardized mortality rate (ASMR), age-standardized DALY rate (ASDR) and socio-demographic index(SDI) in 195 countries and territories in 1990 and 2017, and their temporal trends from 1990 to 2017.

China	34.06	28.97	34.19	18.83	787.33	391.66	-0.73	-2.27	-2.76	0.71	0.59
Colombia	23.68	15.89	24.38	10.68	514.86	239.55	-1.84	-3.39	-3.07	0.63	0.55
Comoros	10.61	6.20	11.17	6.61	270.43	151.04	-2.21	-2.17	-2.38	0.43	0.35
Costa Rica	38.53	30.85	34.37	17.24	707.84	359.69	-1.37	-3.22	-3.13	0.66	0.60
Croatia	22.73	12.51	20.96	8.91	457.56	182.02	-1.99	-2.98	-3.28	0.83	0.77
Cuba	7.38	6.27	7.57	5.21	157.40	110.49	-0.47	-1.25	-1.20	0.69	0.63
Cyprus	8.02	8.50	8.30	6.19	172.23	125.39	0.75	-0.85	-0.92	0.87	0.81
Czech Republic	17.78	8.59	17.86	5.98	371.29	124.20	-2.63	-4.08	-4.01	0.85	0.80
Democratic Republic of the Congo	9.80	7.12	10.45	7.59	235.43	171.80	-1.20	-1.20	-1.19	0.36	0.29
Denmark	10.43	6.81	8.79	4.92	183.00	96.21	-1.36	-2.03	-2.27	0.92	0.89
Djibouti	9.15	6.05	9.72	6.47	228.85	147.27	-1.88	-1.85	-1.99	0.49	0.39
Dominica	20.60	15.54	21.92	15.87	464.90	334.89	-1.14	-1.31	-1.32	0.69	0.62
Dominican Republic	6.84	7.13	7.30	7.13	158.62	164.56	0.01	-0.20	-0.10	0.59	0.51
Ecuador	26.74	17.28	28.96	17.97	601.43	355.38	-1.51	-1.70	-1.78	0.64	0.57
Egypt	4.60	3.51	4.94	3.74	105.95	78.71	-1.06	-1.11	-1.07	0.60	0.53
El Salvador	13.17	16.70	13.86	13.49	300.49	288.98	0.44	-0.55	-0.67	0.59	0.50
Equatorial Guinea	14.74	4.50	15.41	4.84	375.76	103.70	-5.05	-4.93	-5.45	0.63	0.42
Eritrea	15.86	9.11	16.53	9.57	428.73	238.67	-2.25	-2.18	-2.38	0.41	0.32
Estonia	27.42	18.69	25.56	10.82	614.79	244.01	-1.30	-3.37	-3.71	0.86	0.79
Ethiopia	14.20	5.48	14.58	5.79	370.21	133.76	-3.91	-3.78	-4.19	0.33	0.21
Federated States of Micronesia	15.03	11.36	15.85	12.05	364.72	266.04	-1.07	-1.05	-1.19	0.58	0.53
Fiji	6.30	5.47	6.76	5.85	144.99	123.30	-0.59	-0.62	-0.61	0.64	0.59
Finland	16.78	7.50	12.84	4.66	261.71	93.14	-3.21	-3.83	-3.83	0.89	0.85
France	11.19	7.17	10.91	4.98	207.30	98.21	-1.59	-2.93	-2.72	0.87	0.82
Gabon	9.56	5.68	10.14	6.07	234.17	135.92	-1.99	-1.96	-2.06	0.65	0.54
Gambia	7.24	5.66	7.87	6.24	159.85	121.03	-0.75	-0.70	-0.86	0.41	0.33
Georgia	14.80	14.20	14.91	14.33	372.47	337.17	0.71	0.72	0.54	0.70	0.65
Germany	19.29	11.81	14.45	6.85	297.10	138.52	-2.16	-2.96	-3.01	0.87	0.83
Ghana	11.07	7.67	11.96	8.36	244.78	164.04	-1.47	-1.44	-1.59	0.54	0.45
Greece	12.03	10.37	11.11	7.46	226.67	153.83	-0.42	-1.48	-1.42	0.82	0.78
Greenland	19.98	11.40	20.71	11.71	484.11	251.75	-2.39	-2.40	-2.74	0.76	0.71
Grenada	13.31	8.16	14.11	8.41	309.98	183.60	-1.50	-1.61	-1.61	0.64	0.57
Guam	5.85	5.27	6.15	4.95	125.70	110.95	-0.96	-1.34	-0.90	0.79	0.75
Guatemala	20.97	19.37	22.67	19.39	471.40	409.17	-0.82	-1.11	-1.09	0.52	0.43
Guinea	14.25	14.52	14.96	15.29	344.48	348.46	0.31	0.30	0.29	0.33	0.24
Guinea-Bissau	21.74	15.18	22.83	16.21	513.53	345.90	-1.02	-0.95	-1.16	0.35	0.26
Guyana	10.08	6.55	10.67	6.83	239.34	156.24	-1.46	-1.53	-1.38	0.58	0.52
Haiti	23.05	15.12	24.38	16.22	556.71	352.22	-1.55	-1.49	-1.68	0.44	0.39
Honduras	8.01	7.07	8.38	7.27	188.35	144.91	-0.62	-0.70	-1.14	0.51	0.43

Table. S1 (Related to Table 1) The age-standardized incidence rate (ASIR), age-standardized mortality rate (ASMR), age-standardized DALY rate (ASDR) and socio-demographic index(SDI) in 195 countries and territories in 1990 and 2017, and their temporal trends from 1990 to 2017.

Hungary	18.92	8.41	19.91	8.28	419.46	174.59	-2.79	-3.04	-3.04	0.82	0.76
Iceland	17.31	8.84	13.20	5.00	265.41	95.40	-2.56	-3.72	-3.91	0.91	0.86
India	10.74	7.46	11.07	7.75	276.08	187.48	-1.45	-1.43	-1.53	0.55	0.42
Indonesia	10.56	7.45	11.09	8.03	257.10	166.48	-1.20	-1.10	-1.52	0.65	0.56
Iran	20.50	14.61	21.88	14.91	472.64	296.79	-0.91	-1.07	-1.34	0.70	0.62
Iraq	7.15	2.79	7.48	2.91	181.97	68.37	-3.89	-3.89	-4.09	0.59	0.50
Ireland	13.31	9.78	13.11	6.24	255.17	114.79	-1.21	-2.93	-3.20	0.88	0.83
Israel	10.30	6.63	10.66	5.75	206.04	114.16	-2.13	-2.80	-2.72	0.82	0.78
Italy	20.93	12.70	19.25	7.70	372.65	147.67	-1.82	-3.49	-3.47	0.84	0.81
Ivory Coast	6.74	5.46	7.17	5.88	150.42	119.42	-0.80	-0.74	-0.90	0.41	0.34
Jamaica	12.98	9.24	13.85	9.21	276.34	197.03	-1.59	-1.83	-1.57	0.68	0.62
Japan	58.76	29.56	29.94	14.22	648.59	275.27	-2.68	-2.85	-3.29	0.87	0.84
Jordan	7.27	4.16	7.55	4.32	178.85	84.07	-2.23	-2.20	-2.99	0.70	0.62
Kazakhstan	29.08	13.04	29.60	13.00	743.07	308.73	-2.93	-2.98	-3.25	0.74	0.67
Kenya	9.91	9.04	10.45	9.55	240.50	219.67	-0.21	-0.20	-0.21	0.50	0.42
Kiribati	14.81	11.31	15.54	11.85	361.79	277.25	-0.73	-0.74	-0.70	0.43	0.39
Kuwait	4.28	2.27	4.39	2.05	92.80	41.63	-2.18	-2.62	-2.76	0.79	0.70
Kyrgyzstan	28.28	13.99	28.33	14.22	757.82	350.13	-2.50	-2.45	-2.78	0.61	0.57
Laos	18.49	8.52	19.28	9.09	464.38	199.34	-2.99	-2.90	-3.26	0.52	0.40
Latvia	25.40	17.21	23.87	12.11	584.56	278.31	-1.42	-2.64	-2.96	0.83	0.77
Lebanon	11.61	7.73	12.06	6.00	275.46	125.33	-1.58	-2.70	-3.08	0.73	0.63
Lesotho	10.32	9.56	11.04	10.17	251.83	235.15	0.29	0.27	0.37	0.49	0.42
Liberia	12.77	10.65	13.62	11.68	288.04	232.10	-0.59	-0.46	-0.74	0.33	0.24
Libya	6.88	6.43	7.26	6.41	162.22	142.81	-0.25	-0.50	-0.54	0.76	0.74
Lithuania	25.49	17.39	24.10	12.81	580.17	298.18	-1.38	-2.48	-2.62	0.84	0.78
Luxembourg	13.64	8.90	12.23	5.35	242.83	99.72	-1.81	-3.30	-3.55	0.92	0.88
Macedonia	18.46	11.26	19.11	11.38	450.45	265.47	-2.20	-2.29	-2.36	0.75	0.69
Madagascar	9.25	6.62	9.70	7.00	239.07	168.88	-1.29	-1.26	-1.35	0.33	0.29
Malawi	5.16	3.25	5.69	3.58	113.88	72.60	-2.38	-2.38	-2.40	0.35	0.26
Malaysia	10.99	7.11	12.04	7.15	242.79	137.02	-2.13	-2.41	-2.54	0.76	0.67
Maldives	7.50	2.43	7.96	2.37	179.05	44.70	-4.67	-4.96	-5.64	0.66	0.54
Mali	22.93	14.61	23.75	15.40	575.48	350.25	-1.66	-1.59	-1.85	0.27	0.19
Malta	11.78	8.18	11.78	6.15	227.73	116.35	-1.61	-2.69	-2.78	0.84	0.79
Marshall Islands	18.24	15.06	19.19	15.75	439.31	365.58	-0.73	-0.76	-0.70	0.55	0.49
Mauritania	14.73	9.63	15.52	10.50	336.40	200.79	-1.32	-1.19	-1.68	0.47	0.38
Mauritius	12.84	6.79	13.60	6.55	291.67	136.60	-2.24	-2.56	-2.62	0.72	0.64
Mexico	11.10	10.06	11.55	7.28	235.47	160.37	-0.65	-1.97	-1.64	0.63	0.58
Moldova	18.10	9.88	17.70	8.89	472.32	231.28	-2.00	-2.30	-2.41	0.68	0.61
Mongolia	59.47	35.62	62.84	37.65	1427.50	842.92	-2.44	-2.42	-2.55	0.66	0.60
Montenegro	7.36	6.17	7.61	6.18	166.77	128.38	-0.87	-0.99	-1.22	0.79	0.73
Morocco	5.18	3.79	5.56	4.09	119.13	82.10	-1.20	-1.17	-1.43	0.58	0.49
Mozambique	8.17	6.66	8.91	7.22	188.89	156.85	-0.66	-0.69	-0.57	0.34	0.22

Table. S1 (Related to Table 1) The age-standardized incidence rate (ASIR), age-standardized mortality rate (ASMR), age-standardized DALY rate (ASDR) and socio-demographic index(SDI) in 195 countries and territories in 1990 and 2017, and their temporal trends from 1990 to 2017.

Myanmar	22.23	10.02	23.05	10.67	564.86	234.40	-3.07	-2.96	-3.38	0.56	0.43
Namibia	5.53	3.10	6.05	3.46	127.87	66.70	-2.57	-2.48	-2.90	0.62	0.53
Nepal	11.24	7.63	11.65	8.14	289.65	183.41	-1.35	-1.24	-1.63	0.43	0.32
Netherlands	15.59	11.32	13.27	6.90	255.00	124.65	-1.21	-2.57	-2.84	0.91	0.87
New Zealand	11.93	7.84	9.86	4.56	203.45	93.43	-1.84	-3.10	-3.15	0.84	0.80
Nicaragua	12.50	9.44	12.89	7.96	293.16	179.48	-1.27	-2.03	-2.05	0.53	0.45
Niger	14.63	11.92	15.66	13.00	338.03	260.60	-0.58	-0.50	-0.83	0.19	0.14
Nigeria	5.57	4.01	6.18	4.49	117.69	81.19	-1.40	-1.36	-1.60	0.49	0.41
North Korea	20.82	20.35	21.32	20.56	524.22	496.79	0.06	0.02	-0.02	0.54	0.51
Northern Mariana Islands	10.07	7.33	10.55	6.59	214.88	134.04	-1.52	-2.09	-2.09	0.76	0.76
Norway	13.84	7.10	11.08	4.51	221.16	85.35	-2.57	-3.46	-3.66	0.91	0.87
Oman	13.22	7.05	13.85	6.83	314.94	140.39	-2.33	-2.57	-3.02	0.74	0.62
Pakistan	5.53	5.22	5.77	5.54	135.71	123.28	-0.46	-0.39	-0.62	0.49	0.39
Palestine	8.92	5.16	9.25	5.38	223.19	121.36	-1.83	-1.82	-2.05	0.54	0.44
Panama	14.16	14.98	13.34	8.74	293.96	191.28	-0.18	-1.70	-1.68	0.68	0.60
Papua New Guinea	18.91	15.96	18.91	16.15	507.99	417.28	-0.45	-0.41	-0.53	0.42	0.37
Paraguay	10.49	8.10	11.37	8.44	237.25	180.62	-1.37	-1.50	-1.43	0.62	0.54
Peru	19.39	14.12	20.81	14.16	446.36	298.86	-1.47	-1.73	-1.73	0.64	0.57
Philippines	6.57	3.98	7.03	4.19	152.35	91.15	-2.00	-2.04	-2.05	0.62	0.56
Poland	16.68	8.66	17.59	8.85	399.12	193.77	-2.35	-2.55	-2.65	0.84	0.77
Portugal	25.75	15.70	26.26	11.54	544.46	241.95	-1.88	-3.21	-3.10	0.78	0.72
Puerto Rico	10.54	6.38	10.70	4.26	206.19	85.18	-2.01	-3.73	-3.63	0.81	0.75
Qatar	10.15	5.61	11.08	5.15	213.22	95.70	-2.54	-3.23	-3.28	0.77	0.70
Republic of Congo	13.04	7.34	13.63	7.80	331.65	179.03	-2.40	-2.32	-2.59	0.57	0.47
Romania	13.81	9.54	14.15	9.48	344.73	219.25	-1.52	-1.61	-1.86	0.78	0.72
Russia	30.01	17.83	27.92	13.10	684.91	308.43	-2.13	-3.06	-3.28	0.79	0.74
Rwanda	12.99	5.51	13.72	5.95	327.32	131.86	-3.96	-3.83	-4.23	0.41	0.32
Saint Lucia	16.90	10.16	17.99	10.19	390.21	218.60	-2.12	-2.35	-2.37	0.65	0.59
Saint Vincent and the Grenadines	13.09	9.37	13.91	9.58	300.33	217.17	-1.29	-1.42	-1.33	0.61	0.54
Samoa	11.51	10.10	12.10	10.81	280.27	227.66	-0.55	-0.47	-0.85	0.58	0.55
Sao Tome and Principe	14.45	15.52	15.79	17.10	316.97	332.22	0.21	0.23	0.12	0.49	0.38
Saudi Arabia	6.99	4.82	7.64	4.74	150.47	88.66	-1.15	-1.45	-1.66	0.78	0.63
Senegal	14.50	11.97	15.57	13.06	327.61	259.38	-0.43	-0.37	-0.62	0.37	0.31
Serbia	12.39	8.71	12.68	8.12	298.55	178.09	-1.09	-1.63	-1.91	0.75	0.69
Seychelles	10.98	5.68	11.41	5.54	270.31	123.65	-2.57	-2.77	-3.02	0.69	0.63
Sierra Leone	13.46	12.19	14.50	13.32	309.01	268.00	0.06	0.12	-0.10	0.36	0.26
Singapore	19.58	10.17	17.67	5.09	364.58	97.51	-2.53	-4.90	-5.14	0.87	0.82
Slovakia	21.18	13.89	16.81	7.93	377.34	167.06	-1.40	-2.65	-2.82	0.84	0.78

Table. S1 (Related to Table 1) The age-standardized incidence rate (ASIR), age-standardized mortality rate (ASMR), age-standardized DALY rate (ASDR) and socio-demographic index(SDI) in 195 countries and territories in 1990 and 2017, and their temporal trends from 1990 to 2017.

Slovenia	20.58	11.80	20.08	8.21	434.81	162.96	-2.18	-3.58	-3.93	0.86	0.81
Solomon Islands	16.53	12.51	17.04	13.03	424.91	312.54	-0.91	-0.88	-1.00	0.43	0.37
Somalia	11.84	8.71	12.50	9.24	296.25	219.23	-1.55	-1.51	-1.57	0.24	0.18
South Africa	6.37	4.35	6.59	4.63	161.61	100.87	-1.81	-1.68	-2.18	0.68	0.62
South Korea	49.24	29.15	47.08	14.09	1185.41	297.43	-1.97	-4.88	-5.25	0.87	0.81
South Sudan	10.62	7.96	11.31	8.44	260.80	197.83	-1.40	-1.41	-1.38	0.28	0.22
Spain	19.29	12.30	14.57	6.79	298.26	136.40	-1.59	-2.70	-2.84	0.83	0.78
Sri Lanka	7.95	4.27	8.58	4.06	180.63	82.39	-2.32	-2.74	-2.86	0.68	0.58
Sudan	17.61	13.94	18.58	14.91	427.58	322.16	-0.81	-0.76	-1.00	0.48	0.34
Suriname	8.75	6.07	9.36	6.38	201.81	138.01	-1.37	-1.43	-1.46	0.64	0.58
Swaziland	9.74	7.27	10.45	7.80	233.09	178.61	-0.51	-0.54	-0.33	0.58	0.51
Sweden	11.77	5.40	8.93	3.78	175.77	70.86	-2.85	-3.05	-3.22	0.88	0.84
Switzerland	12.42	6.61	9.08	3.81	182.90	73.13	-2.36	-3.17	-3.34	0.89	0.86
Syria	5.96	4.10	6.37	4.27	138.81	87.89	-1.74	-1.84	-2.03	0.61	0.50
Taiwan (Province of China)	19.08	15.84	16.30	9.17	361.04	182.66	-0.82	-2.16	-2.49	0.86	0.79
Tajikistan	23.25	17.35	23.75	17.83	600.21	438.35	-1.13	-1.09	-1.25	0.52	0.48
Tanzania	8.67	5.76	9.21	6.13	210.22	138.13	-1.87	-1.85	-1.94	0.41	0.33
Thailand	8.69	4.80	9.14	4.38	209.09	102.35	-2.58	-3.07	-3.03	0.68	0.60
Timor-Leste	12.78	8.14	13.57	8.83	309.08	181.22	-1.79	-1.71	-2.11	0.51	0.38
Togo	13.42	11.28	14.40	12.26	308.12	251.65	-0.35	-0.30	-0.48	0.41	0.33
Tonga	17.20	13.58	18.54	14.44	378.42	304.40	-0.87	-0.93	-0.74	0.63	0.58
Trinidad and Tobago	10.09	4.52	10.90	4.60	218.58	91.99	-3.54	-3.80	-3.78	0.70	0.66
Tunisia	6.88	5.00	7.23	5.01	140.10	97.08	-1.43	-1.59	-1.61	0.68	0.58
Turkey	20.49	9.87	20.67	9.24	525.64	216.08	-2.88	-3.13	-3.50	0.73	0.62
Turkmenistan	19.14	9.66	19.68	9.79	485.96	238.86	-2.76	-2.79	-2.91	0.70	0.63
Uganda	7.60	5.13	8.13	5.51	183.83	124.44	-2.01	-1.98	-2.05	0.39	0.26
UK	15.54	10.33	12.62	5.99	241.48	109.69	-1.82	-2.99	-3.18	0.84	0.79
Ukraine	28.36	17.82	23.42	12.18	617.82	321.39	-2.00	-2.77	-2.91	0.74	0.70
United Arab Emirates	9.31	8.45	10.17	8.76	201.61	174.55	-0.43	-0.63	-0.63	0.80	0.74
Uruguay	19.73	13.36	20.83	13.51	445.99	282.03	-1.37	-1.51	-1.61	0.71	0.65
USA	7.66	6.00	5.51	3.22	113.98	68.87	-1.30	-2.20	-2.11	0.87	0.82
Uzbekistan	17.75	10.83	18.00	10.93	463.11	268.42	-1.44	-1.45	-1.64	0.63	0.55
Vanuatu	15.56	13.08	16.48	14.00	383.57	314.56	-0.62	-0.58	-0.71	0.48	0.43
Venezuela	17.98	13.95	18.23	9.68	380.13	208.36	-1.06	-2.55	-2.41	0.66	0.60
Vietnam	17.82	8.98	18.34	8.94	454.70	211.92	-3.19	-3.31	-3.39	0.61	0.51
Virgin Islands, U.S.	10.77	9.96	11.35	8.56	239.20	183.36	-0.05	-1.05	-0.98	0.81	0.75
Yemen	21.25	18.49	22.01	19.57	531.30	436.93	-0.62	-0.53	-0.86	0.43	0.32
Zambia	11.97	6.87	12.65	7.31	298.64	168.40	-2.76	-2.72	-2.89	0.47	0.37
Zimbabwe	10.38	10.47	11.11	11.14	244.69	255.52	0.80	0.75	0.99	0.46	0.44

Table. S1 (Related to Table 1) The age-standardized incidence rate (ASIR), age-standardized mortality rate (ASMR), age-standardized DALY rate (ASDR) and socio-demographic index(SDI) in 195 countries and territories in 1990 and 2017, and their temporal trends from 1990 to 2017.
